# Real-time PCR for detection of *Streptococcus suis* serotype 2 in cerebrospinal fluid of human patients with meningitis

**DOI:** 10.1016/j.diagmicrobio.2010.12.015

**Published:** 2011-08

**Authors:** Tran Vu Thieu Nga, Ho Dang Trung Nghia, Le Thi Phuong Tu, To Song Diep, Nguyen Thi Hoang Mai, Tran Thi Hong Chau, Dinh Xuan Sinh, Nguyen Hoan Phu, Tran Thi Thu Nga, Nguyen Van Vinh Chau, James Campbell, Ngo Thi Hoa, Nguyen Tran Chinh, Tran Tinh Hien, Jeremy Farrar, Constance Schultsz

**Affiliations:** a Oxford University Clinical Research Unit, Hospital for Tropical Diseases, Ho Chi Minh City, Vietnam; b Hospital for Tropical Diseases, Ho Chi Minh City, Vietnam; c Department of Infectious diseases, Pham Ngoc Thach University of Medicine, Ho Chi Minh City, Vietnam; d Center for Poverty-related Communicable Diseases (CPCD), Amsterdam Institute for Global Health and Development, Academic Medical Center, University of Amsterdam, the Netherlands; e Department of Medical Microbiology, Academic Medical Center, University of Amsterdam, the Netherlands

**Keywords:** Bacterial meningitis, *Streptococcus suis* serotype 2, CSF, Real-time PCR

## Abstract

*Streptococcus suis* serotype 2 is an emerging zoonotic pathogen and is the main cause of acute bacterial meningitis in adult patients in Vietnam. We developed an internally controlled real-time PCR for detection of *S. suis* serotype 2 in cerebrospinal fluid (CSF) samples targeted at the *cps2J* gene. Sensitivity and specificity in culture-confirmed clinical samples were 100%. The PCR detected *S. suis* serotype 2 infection in 101 of 238 (42.4%) prospectively collected CSF samples, of which 55 (23%) were culture positive. Culture-negative but PCR-positive CSF samples were significantly associated with the use of antimicrobial agents before admission. *S. suis* serotype 2 infection was more common than infections with *Streptococcus pneumoniae* and *Neisseria meningitidis* combined. Our results strikingly illustrate the additional diagnostic value of PCR in patients who are pretreated with antimicrobial agents and demonstrate the extremely high prevalence of *S. suis* infections among Vietnamese adult patients with bacterial meningitis.

## 1 Introduction

*Streptococcus suis* is an emerging zoonotic human pathogen. *S. suis* infection is acquired through exposure to contaminated pigs or pig meat. Healthy pigs can carry multiple serotypes of *S. suis* in their nasal cavities, tonsils, and upper respiratory, genital, and alimentary tracts. Based on differences in antigenic properties of the polysaccharide capsule, 33 serotypes have been distinguished to date, of which only a limited number are responsible for infections in pigs, including serotypes 1 to 9 and 14. Serotype 2 is considered to be the most pathogenic for both humans and pigs and is the single most common serotype found in human infection (Gottschalk et al., 2007).

Over the past few years, the number of reported *S. suis* infections in humans has increased substantially, with most cases originating in Southeast Asia where there is a high density of pigs. Increased awareness, particularly following the occurrence of an outbreak of human and pig infection in the Sichuan province in China in 2005, has likely contributed to this increase in reported human infections (Tang et al., 2006; Ye et al., 2006). Meningitis and septicemia are the most common clinical manifestations of human *S. suis* infection; hearing loss is a frequent complication (Wertheim et al., 2009a).

Although *S. suis* can be cultured from cerebrospinal fluid (CSF) or blood samples with use of standard microbiological techniques, infection often goes undiagnosed or positive cultures are misidentified as *Streptococcus* species, alpha-hemolytic or viridans streptococci, *Enterococcus faecalis*, *Aerococcus viridans*, or *Streptococcus pneumoniae* (Donsakul et al., 2003; Lutticken et al., 1986). Furthermore, culture results can be negative as a result of antibiotic use before the collection of specimens.

During a randomized placebo-controlled clinical trial on the adjuvant use of dexamethasone in adult patients with bacterial meningitis, carried out at the Hospital for Tropical Diseases in Ho Chi Minh City, Vietnam, *S. suis* serotype 2 was found to be the most common pathogen isolated from CSF cultures (Nguyen et al., 2007). As up to 60% of patients had used antimicrobial agents before submission to the hospital, and culture results were negative for 50% of patients (Nguyen et al., 2007), an internally controlled diagnostic real-time PCR was set up for detection of *S. suis* serotype 2 to further study the importance of this pathogen in patients with bacterial meningitis in this region. Here we report the design of this method and its prospective evaluation.

## 2 Materials and methods

### 2.1 Sample collection

This study was performed at the Hospital for Tropical Diseases, a tertiary referral hospital for infectious diseases. CSF samples were collected and stored as part of a randomized placebo-controlled clinical trial carried out at the Hospital for Tropical Diseases between November 1996 and May 2005 (Nguyen et al., 2007). CSF samples were sent for biochemical and microbiological investigations, and an aliquot was stored at −70 °C in a dedicated freezer on the ward. These samples were studied retrospectively. CSF samples were prospectively collected from all consecutive adult patients (age ≥15 years) presenting with fever and neck stiffness and/or altered consciousness at the Hospital for Tropical Diseases from May 2006 until June 2009, and were aliquoted immediately after lumbar puncture on the ward. Aliquots were sent to the biochemistry and microbiology laboratories for immediate processing and analyses. A separate aliquot was sent to the molecular diagnostics laboratory, where samples where stored for a maximum of 48 h at −70 °C until testing (prospective study). Standard measures for prevention of PCR contamination are operational at the molecular diagnostic laboratory, including a unidirectional workflow in physically separated laboratories for reagent preparation, nucleic acid extraction, and amplification and analysis, respectively.

Demographic, clinical, and laboratory data were recorded for all patients. The study was approved by the ethical review boards of the Hospital for Tropical Diseases and the University of Oxford (OXTREC).

### 2.2 Bacterial culture

All CSF samples were spun down and a Gram stain was made. The pellet was inoculated on blood and chocolate agar plates and in brain heart infusion broth for enrichment. Plates were incubated at 37 °C in 5% CO_2_ for 18 h. The broth was incubated aerobically and subcultured if growth was present. Bacteria were identified using standard identification methods. *S. suis* was identified on the basis of colony morphology, negative catalase reaction, optochin resistance, and APIStrep (Biomerieux, Ho Chi Minh City, Vietnam). Serotyping was performed by slide agglutination with use of specific antisera (Statens Serum Institute, Copenhagen, Denmark).

Blood culture was performed using the BACTEC 9050 system, and positive culture results were identified as described above.

### 2.3 Primers and probes

Primers and probe for *S. suis* serotype 2 real-time PCR were designed using Primer Express Software and BLAST analysis, and were targeted at the *cps2J* gene (Smith et al., 1999, 2000), which is part of the operon encoding the serotype 2 and serotype 1/2 specific polysaccharide capsule of *S. suis*. Primers cps2JF (GGTTACTTGCTACTTTTGATGGAAATT) and cps2JR (CGCACCTCTTTTATCTCTTCCAA) and probe (FAM-TCAAGAATCTGAGCTGCAAAAGTGTCAAATTGA-TAMRA) were used for amplification and detection of an 88-bp amplicon. Primers and probes for real-time PCR for detection of *S. pneumoniae*, *Haemophilus influenzae* type b, and *N. meningitidis* were as described by Corless et al. (2001) except that for all probes, FAM (6-carboxyfluorescein) and TAMRA were used as reporter and quencher, respectively.

Primers and probe for detection of internal control (IC) DNA (see below) were as described by van Doornum et al. (2003). The IC probe was labeled with Cy5 and BHQ1.

### 2.4 Internal control

The efficiency of the DNA extraction and the amplification during the PCR was monitored using an IC, consisting of a pretest determined concentration of Phocid herpesvirus. IC was added to all samples before DNA extraction, as described by van Doornum et al. (2003). Concentration of IC was such that after efficient extraction and amplification, a Cy5 cycle threshold value (Ct value) between 33 and 37 should be expected for the IC-specific PCR reaction. Higher Ct values or negative results were interpreted as loss of DNA during extraction or inhibition of the PCR assay, in which case extraction and amplification were repeated. PhHV was kindly provided by M. Schutten (EMC, Rotterdam, the Netherlands).

### 2.5 DNA extraction of pure cultures and CSF samples

A 100-μL aliquot of a bacterial suspension or of unspun CSF was treated with 0.1 volume of prelysis buffer (1% SDS, 5% Tween 20, and 5% Sarkosyl in 1× TE) at 37 °C for 1 h. A 20-μL volume of IC at a predetermined concentration was added to the sample, and DNA was extracted by manual extraction (retrospective study) or automated extraction using the EasyMag extraction system (BioMerieux, Ho Chi Minh City, Vietnam), according to manufacturer's instructions (prospective study). The manual extraction was performed as described by Boom et al. (1999), using lysis buffer L7 that contains 1 mg/mL α-casein. The DNA was eluted in a final volume of 100 μL.

### 2.6 PCR components and amplification

The final PCR volume was 25 μL. The PCR mix consisted of 5 mmol/L MgCl_2_, 0.2 mmol/L each deoxynucleoside triphosphates dATP, dCTP, dGTP, dUTP, and 1 U of Hot Start Taq DNA polymerase (Qiagen, Hanoi, Vietnam) to which 5 μL of extracted DNA was added. Final concentrations of the 2 primers and probe sets for target and IC were 0.4 μmol/L of each primer and 0.1 μmol/L of each probe. PCR amplification conditions consisted of 15 min at 95 °C and 45 cycles of 30 s at 95 °C, 30 s at 60 °C, and 30 s at 72 °C in a Chromo 4 Real-time PCR system (Biorad, Ho Chi Minh City, Vietnam). Negative (no-template) controls of both extraction and PCR were included in each run.

The PCR was considered positive if negative controls were all negative and a FAM signal with a Ct value of ≤40 could be obtained from the sample. A PCR was considered negative if negative controls were all negative, and the IC showed a Cy5 Ct value within the expected range, and a FAM signal could not be obtained from the sample or the Ct value was >40. Any PCR that yielded a FAM Ct value >35 was repeated in duplicate for confirmation. The PCR was considered indeterminate if the IC showed a Cy5 Ct value outside of the expected range and a FAM signal could not be obtained or the Ct value was >40, in which case DNA extraction and the PCR were repeated as described before.

The sensitivity and specificity of the PCR assay were determined on the basis of PCR results in bacterial culture-confirmed samples. PCR for *S. suis* serotype 2 was run on all culture-positive samples, including those growing *S. suis*, *S. pneumoniae*, *N. meningitidis*, *H. influenzae*, or other pathogens (Tables 2 and 3).

### 2.7 Analytical sensitivity

To determine the detection limit of the assay, including the DNA extraction, a 10-fold serial dilution of a 0.5 McFarland suspension of *S. suis* serotype 2 strain 31533, kindly provided by M. Gottschalk (Montreal, Canada), was prepared in Todd Hewitt Broth. Fifty microliters of each dilution was spread out on blood agar plates in triplicate and incubated at 37 °C in 5% CO_2_ overnight for colony counting. A 100-μL volume of each dilution was used for DNA extraction in triplicate, in the absence and presence of IC. Five microliters of DNA was used for real-time PCR, as described above.

## 3 Results

The PCR assay detected *S. suis* serotype 2 at a concentration of 2 × 10^2^ colony-forming units (CFUs) per milliliter, resulting in an analytical sensitivity of 1–5 CFUs per reaction. This analytical sensitivity did not vary in the presence or absence of IC DNA (Table 1).

Sensitivity of the PCR was 100% when tested against 114 stored samples from culture-confirmed cases of meningitis with *S. suis* serotype 2 (Table 2A). The PCR was negative in all 99 samples that were culture-positive for other bacterial pathogens, including *S. suis* of other serotypes (100% specificity, Table 2A). All PCR-negative samples gave Ct values for the IC within the expected range.

Stored CSF samples that were culture positive for *S. pneumoniae*, *N. meningitidis*, and *H. influenzae* type b were also subjected to real-time PCR for specific detection of these pathogens. Of 50 samples culture positive for *S. pneumoniae*, 48 were positive in the PCR for detection of *S. pneumoniae* DNA, while all 11 samples culture positive for *N. meningitidis* and all 4 samples positive for *H. influenzae* type b were also positive in the respective specific PCRs.

During the study period, we admitted 248 consecutive patients with a clinical suspicion of bacterial meningitis. Demographic, clinical, and outcome characteristics of these 248 patients are shown in Table 3. CSF samples from 238 patients were prospectively studied using bacterial culture and real-time PCR for detection of *S. suis* serotype 2, *N. meningitidis*, *H. influenzae* type b, and *S. pneumoniae*. A lumbar puncture was contraindicated in one patient because of risk of brain herniation. CSF samples were erroneously not sent for PCR analysis for the remaining 9 patients.

All 55 *S. suis* serotype 2 culture-positive samples collected prospectively on admission were also positive in the *S. suis* serotype 2-specific PCR. The admission CSF sample of one patient with culture-confirmed *S. suis* serotype 2 meningitis was not available for PCR analysis and PCR result of the second CSF sample from this patient (collected after 5 days of antibiotic treatment) was negative. The *S. suis* serotype 2 specific PCR was negative in all 39 samples, which were culture confirmed with other bacterial pathogens, including *S. suis* of other serotypes (Table 2B).

*S. suis* was the most commonly identified pathogen (Table 4). PCR for *S. suis* serotype 2 was positive in 101 of 238 (42.4%) samples, of which 55 (23.1%) were culture positive. *S. pneumoniae* and *N. meningitidis* were detected in 37 (15.5%) and 11 (4.6%) patients of which 16 (6.7%) and 4 (1.7%) were culture positive, respectively. *Listeria* species were cultured from CSF of 4 patients. Infections with multiple bacterial species were not detected. All samples gave the expected results for the IC. Bacterial pathogens were detected in 183 of 248 (73.8%) adult patients suspected of bacterial meningitis when combining results of Gram stain, bacterial culture and PCR on CSF, and blood culture.

CSF was significantly more often culture negative in patients who were pretreated with antimicrobial agents before admission (Table 5). In contrast, detection rates by PCR were similar in patients who were pretreated and those who were not, although Ct values were significantly higher in patients who had received antimicrobial agents before collection of the CSF sample (Table 5). We compared characteristics between patients who were only positive by PCR in CSF and those of whom CSF or blood samples were also culture positive (Table 6). Patient characteristics related to exposure and clinical presentation were highly similar between the two groups, with the exception of a higher age and a higher prevalence of diabetes mellitus in patients who were PCR-positive only. In contrast, the median duration of illness was significantly longer and pretreatment with antimicrobial agents significantly more common in patients who were PCR-positive only. This was also reflected by lower CSF neutrophil counts, higher CSF glucose levels, and lower CSF lactate levels in the latter patients (Table 6).

## 4 Discussion

Human infections with *S. suis* are increasingly reported from various geographical areas. *S. suis* serotype 2 is the most common pathogen detected in adult patients with acute bacterial meningitis in Vietnam (Mai et al., 2008; Wertheim et al., 2009b). While *S. suis* is not difficult to culture on blood agar plates supplemented with 5% CO_2_, CSF cultures may remain negative because of prior use of antimicrobial agents or low bacterial load. We developed a highly sensitive and specific real-time PCR for detection of *S. suis* serotype 2 in CSF. We designed primers targeted at the *cps2J* gene, which encodes a putative glycosyl transferase involved in the formation of the serotype 2 capsular polysaccharide. This gene was also used by other investigators as a target for conventional PCR for specific detection of *S. suis* serotype 2 in tonsillar and other pig samples (Wisselink et al., 2002). The *cps2J* gene is present in strains of serotype 2 and of serotype 1/2. Serotyping confirmed the presence of *S. suis* serotype 2 in all culture-positive CSF samples, and none contained serotype 1/2. To our knowledge, *S. suis* serotype 1/2 infection has never been reported in humans. So far, serotype 2 is the cause of more than 95% of reported human *S. suis* infections (Wertheim et al., 2009a), and only sporadic single cases of patients infected with *S. suis* serotypes 1, 4, and 16 have been described (Nghia et al., 2008; Wertheim et al., 2009a). However, one patient in our retrospective analysis and 2 patients in the prospective analysis were infected with *S. suis* serotype 14. As expected, these samples were negative in the PCR. While the absolute number of patients reported with *S. suis* serotype 14 infection is still very low, this serotype appears to contribute consistently to the infectious burden of *S. suis* as reported in Thailand (Kerdsin et al., 2009) and also observed in our study. While serotype 2 by far remains the predominant strain associated with human infection at present, inclusion of additional primer sets for detection of serotype 14 or generic detection of *S. suis* may therefore be considered in the future.

Our assay showed 100% sensitivity against samples that were culture positive for *S. suis* serotype 2, while analysis of CSF samples that were culture or PCR-positive for other pathogens than *S. suis* serotype 2, including *S. pneumoniae*, *N. meningitidis*, and *H. influenzae*, indicated 100% specificity of the assay. Thus, in a tertiary referral setting in southern Vietnam, the positive and negative predictive values of the test are 100%. Results of real-time PCR for specific detection of *S. pneumoniae*, *N. meningitidis*, and *H. influenzae* on samples studied retrospectively, which had been culture positive for these pathogens, showed that bacterial DNA was still detectable in 97% of samples indicating that the bacterial DNA was not affected by storage.

Prospective evaluation showed a striking additional diagnostic value of the *S. suis* serotype 2 PCR over culture, further strengthening observations that the prevalence of *S. suis* infections among Vietnamese patients with bacterial meningitis is extremely high. The diagnostic yield increased by 84% for patients with *S. suis* serotype 2 infection when using PCR. Similar differences in detection rates between culture and PCR were found for *S. pneumoniae* and *N. meningitidis*. In large part, these differences can be explained by the use of antimicrobial agents before admission and collection of specimens. In our study, overall 63.3% of patients had received antibiotics before admission, and this proportion was significantly higher in patients with culture-negative CSF samples. Furthermore, bacterial loads as assessed by Ct values in our PCR were significantly lower in pretreated patients. However, while PCR is clearly an important tool in the diagnosis of bacterial meningitis, it should not replace Gram stain and bacterial culture, given the need of a rapid presumptive diagnosis and antimicrobial susceptibility data in the treatment of this life-threatening disease. In addition, the pathogens that can be detected using PCR generally do not cover the full spectrum of potential causes of bacterial meningitis, indicating the continued need of bacterial culture.

*S. suis* was detected at much higher rates than *S. pneumoniae* and *N. meningitidis* in our study population. *S. suis* is increasingly recognized as an important cause of bacterial meningitis in adults, not only in Vietnam but also in China, Thailand, Singapore, and other countries in the region, while sporadic cases are reported worldwide. Risk factors for *S. suis* infection include (occupational) exposure to pigs and pig products. Consumption of undercooked pork products is increasingly being suggested as an additional risk factor for *S. suis* infection (Wertheim et al., 2009a). The mortality of *S. suis* infection varies with the clinical presentation, with the lowest mortality in patients with meningitis and the highest mortality in those presenting with a streptococcal toxic shock-like syndrome (Wertheim et al., 2009a). Resistance to penicillin in *S. suis* is extremely rare. All cultured strains in our study were sensitive to penicillin and ceftriaxone, which are the drugs of choice for treatment of *S. suis* meningitis. *S. suis* meningitis is commonly associated with neurologic sequelae, in particular hearing loss, which can be found in up to 60% of cases (Mai et al., 2008). To reliably determine the burden of disease caused by *S. suis* infection, while taking into account that over-the-counter sales of antimicrobial agents is common in regions where *S. suis* infections predominantly occur, the availability of sensitive and specific diagnostic tools to detect *S. suis* infections is extremely important. To our knowledge, this is the first prospective study on the molecular diagnosis of *S. suis* infections in humans.

In conclusion, we developed a highly sensitive and specific real-time PCR for detection of *S. suis* serotype 2 in CSF, which is now routinely used in a setting where human *S. suis* serotype 2 infection is endemic.

## Figures and Tables

**Table 1:** Comparative analytical sensitivities of *S. suis* serotype 2 primers in the presence and absence of IC DNA

IC DNA	Mean Ct value at the following bacterial concentration		
	10^5^ CFU/mL	10^4^ CFU/mL	10^3^ CFU/mL
Present	29.99	33.25	36.78
Absent	30.21	33.19	36.80

**Table 2:** Sensitivity and specificity of *S. suis* serotype 2 PCR on culture-confirmed CSF samples, retrospective study (A) and prospective study (B)

A.	No. of samples	PCR positive	PCR negative
*S. suis* serotype 2	114	114	0
*S. suis* serotype 14	1	0	1
*N. meningitidis*	11	0	11
*Neisseria* species	1	0	1
*S. pneumoniae*	50	0	50
*H. influenzae*	5	0	5
*Streptococcus bovis*	2	0	2
*Streptococcus agalactiae*	1	0	1
Streptococci, viridans group	6	0	6
*Staphylococcus aureus*	2	0	2
*Escherichia coli*	8	0	8
*Klebsiella pneumoniae*	10	0	10
*Proteus mirabilis*	1	0	1
*Pseudomonas aeruginosa*	1	0	1
Total	213	114	99


B.	No. of samples	No. of PCR positive	No. of PCR negative
*S. suis* serotype 2	55	55	0
*S. suis* serotype 14	2	0	2
*S. suis*, untypeable	1	0	1
*N. meningitidis*	4	0	4
*S. pneumoniae*	16	0	16
*H. influenzae*	1	0	1
*Streptococcus agalactiae*	1	0	1
Nonhemolytic streptococci	1	0	1
*Staphylococcus aureus*	1	0	1
*Escherichia coli*	3	0	3
*Klebsiella pneumoniae*	2	0	2
*Acinetobacter* spp.	1	0	1
*Enterococcus avium*	1	0	1
*Listeria* spp.	4	0	4
*Salmonella* spp.	1	0	1
Total	94	55	39

**Table 3:** Demographic, clinical, and outcome characteristics of 248 patients included in prospective study

Characteristics	Frequency (%) *N* = 248
Age (years), median (interquartile range)	46.5 (34–59)
Sex, male	181 (73.0)
Occupation	
Farmer	79 (31.9)
Worker	35 (14.1)
Student	4 (1.6)
Seller	22 (8.9)
Occupation related to pigs	
✓ Butcher	7 (2.8)
✓ Slaughterer	6 (2.4)
✓ Pig breeder	4 (1.6)
Housewife	14 (5.7)
Other	32 (12.9)
No job	45 (18.2)
Underlying diseases/predisposing factors	
Diabetes mellitus	19 (7.7)
Alcoholism	18 (7.3)
Otitis media	6 (2.4)
Splenectomy	2 (0.8)
Head trauma	26 (10.5)
Cardiovascular diseases^a^	17 (6.9)
Pig/pork exposures^b^	51 (20.6)
HIV/AIDS	4 (1.6)
Used intravenous antibiotics before admission	157 (63.3)

a Valvular heart diseases, atrial fibrillation.

b Occupation related to pig/pork, eating pig's intestines and fresh blood, etc.

**Table 4:** Results of Gram stain, culture, and real-time PCR for detection of *S. suis* serotype 2, *S. pneumoniae*, *H. influenzae* type b, and *N. meningitidis* on CSF samples, and blood culture, prospective study

	No. of positive specimens				
	CSF Gram stain (*n* = 247)	CSF culture (*n* = 247)	CSF PCR (*n* = 238)	Blood culture (*n* = 222)	Total (*N* = 248)
*S. suis* serotype 2	50	61^a^	101^a^	34	107
*S. suis* serotype 14	1	2	0	1	2
*S. suis* untypeable	1	1	0	0	1
*N. meningitidis*	4	4	11	2	11
*S. pneumoniae*	9	16	37^b^	8	39
*H. influenzae*, nontypeable	0	1	0	1	1
*Streptococcus bovis*	1	1	nd^c^	0	1
*Streptococcus agalactiae*	2	1	0	2	2
β-Hemolytic streptococci	0	0	0	1	1
Nonhemolytic streptococci	0	1	0	1	1
*Staphylococcus aureus*	1	1	0	3	3
*Escherichia coli*	1	3	0	2	3
*Klebsiella pneumoniae*	2	4	0^d^	1	4
*Acinetobacter* spp.	0	1	0	0	1
*Enterococcus avium*	0	1	0	0	1
*Listeria* spp.	1	4	0	2	4
*Salmonella* spp.	0	1	0	0	1
Total	73	103	149	58	183

nd = not done.

a CSF samples were unavailable for PCR for 6 patients.

b *S. pneumoniae*-specific PCR was negative in 1 patient; CSF sample unavailable for PCR for 1 patient.

c CSF sample unavailable for PCR.

d CSF sample unavailable for PCR for 2 patients.

**Table 5:** Result of CSF investigations for *S. suis* serotype 2, *S. pneumoniae*, *N. meningitidis*

Antimicrobial agents before admission	Result of CSF investigations for *S. suis* serotype 2, *S. pneumoniae*, *N. meningitidis*			
	Culture positive, no. (%)	PCR positive, no. (%)	Median Ct value^a^ (range)	*P* value^b^
Used	34/98 (34.7)	97/97 (100)	27.78 (15.39–38)	<0.001
Not used	39/47 (82.0)	42/43 (97.7)	24.66 (19.76–31.24)	
Unknown	8/11 (72.7)	10/11 (90.9)	24.57 (20.76–29)	

a Cycle treshold value of real-time PCR.

b Difference between Ct values for antimicrobial agents used versus not used, Wilcoxon rank-sum test.

**Table 6:** Characteristics of patients with *S. suis* meningitis confirmed by CSF and/or blood culture and PCR, or only confirmed by PCR

Characteristics	CSF and/or blood culture and PCR positive (*n* = 67)	PCR positive (*n* = 43)	*P* value^a^
General information			
Age, median (IQR)	48 (38–56)	53 (45–61)	0.026
Male sex (*n*, %)	57 (85.1)	34 (79.1)	0.416
Residence (rural) (*n*, %)	53 (79.1)	32 (74.41)	0.567
Underlying diseases and exposure			
Diabetes mellitus (*n*, %)	1 (1.5)	6 (13.0)	0.010
Alcoholism (*n*, %)	8 (11.9)	2 (4.7)	0.194
Pig exposure (*n*, %)	26 (38.8)	14 (32.6)	0.506
Clinical manifestations			
Days of illness, median (IQR)	3 (3–5)	5 (4–7)	0.004
Antimicrobial therapy before admission (*n*, %)	35 (53.2)	38 (88.4)	<0.001
Fever (*n*, %)	65 (97.0)	42 (97.7)	0.522
Headache (*n*, %)	67 (100)	42 (97.7)	0.391
Nausea/vomiting (*n*, %)	49 (73.1)	27 (62.8)	0.252
Neck stiffness (*n*, %)	61 (91.0)	40 (93.0)	1.000
Glasgow Coma Score, median (IQR)	12 (9–14)	13 (9–15)	0.159
Tinnitus (*n*, %)	50 (74.6)	27 (62.8)	0.118
Deafness (*n*, %)	19 (28.4)	11 (25.6)	0.714
Skin injuries (*n*, %)	18 (26.9)	13 (30.2)	0.702
Herpes labialis (*n*, %)	29 (43.3)	21 (48.8)	0.568
Laboratory investigations median (IQR)			
▪ Blood			
White blood cells (10^3^/L)	18 000 (12 400–23 000)	16 840 (12 600–23 550)	0.941
Neutrophil (%)	88 (83–91)	86.3 (80.3–90)	0.131
▪ CSF			
White cells (10^3^/L)	1570 (760–3480)	1340 (340–2900)	0.153
Neutrophil (%)	86 (74–91)	70 (49–85)	0.002
Protein (g/L)	1.6 (1.3–2)	1.63 (1.2–2)	0.402
CSF/blood glucose ratio	0.24 (0.15–0.31)	0.40 (0.29–0.5)	<0.001
Lactate (mmol/L)	11.2 (6.8–15.7)	5.73 (4.4–8.3)	<0.001
Outcome (survival)	67 (100)	40 (100)^b^	

a Determined using Fisher exact test or Wilcoxon rank-sum test, as appropriate.

b Three patients were transferred to other hospitals and their outcomes are unknown.
